# Prognostic robustness of serum creatinine based AKI definitions in patients with sepsis: a prospective cohort study

**DOI:** 10.1186/s12882-015-0107-4

**Published:** 2015-07-22

**Authors:** Jill Vanmassenhove, Norbert Lameire, Annemieke Dhondt, Raymond Vanholder, Wim Van Biesen

**Affiliations:** Renal Division, Ghent University Hospital, Nephrology section, 0 K12, University Hospital, De Pintelaan 185, B9000 Ghent, Belgium

**Keywords:** AKI, Sepsis, Critical illness, Prognosis, Diagnosis, Mortality

## Abstract

**Background:**

It is unclear how modifications in the way to calculate serum creatinine (sCr) increase and in the cut-off value applied, influences the prognostic value of Acute Kidney Injury (AKI). We wanted to evaluate whether these modifications alter the prognostic value of AKI for prediction of mortality at 3 months, 1 and 2 years.

**Methods:**

We prospectively included 195 septic patients and evaluated the prognostic value of AKI by using three different algorithms to calculate sCr increase: either as the difference between the highest value in the first 24 h after ICU admission and a pre-admission historical (ΔHIS) or an estimated (ΔEST) baseline value, or by subtracting the ICU admission value from the sCr value 24 h after ICU admission (ΔADM). Different cut-off levels of sCr increase (0.1, 0.2, 0.3, 0.4 and 0.5 mg/dl) were evaluated.

**Results:**

Mortality at 3 months, 1 and 2 years in AKI defined as ΔADM > 0.3 mg/dl was 48.1 %, 63.0 % and 63.0 % vs 27.7 %, 39.8 % and 47.6 % in no AKI respectively (OR(95%CI): 2.42(1.06-5.54), 2.58(1.11-5.97) and 1.87(0.81-4.33); 0.3 mg/dl was the lowest cut-off value that was discriminatory. When AKI was defined as ΔHIS > 0.3 mg/dl or ΔEST > 0.3 mg/dl, there was no significant difference in mortality between AKI and no AKI.

**Conclusions:**

The prognostic value of a 0.3 mg/dl increase in sCr, on mortality in sepsis, depends on how this sCr increase is calculated. Only if the evolution of serum creatinine over the first 24 h after ICU admission is taken into account, an association with mortality is found.

**Electronic supplementary material:**

The online version of this article (doi:10.1186/s12882-015-0107-4) contains supplementary material, which is available to authorized users.

## Background

There is growing evidence that Acute Kidney Injury (AKI) is an independent predictor of mortality rather than an innocent bystander. In cardiac surgery it has clearly been demonstrated that even small serum creatinine (sCr) increases are associated with increased mortality risk [1–4]. It remains unclear if this also applies to sepsis.

There is continuing debate on the interpretation of the proposed AKI classification criteria [5–8], and several interpretations of the same AKI definitions continue to appear in the literature [9–12]. The most important issue in this debate relates to the way we calculate the sCr increase. The currently proposed practice to use the highest creatinine value over a certain time span after ICU (Intensive Care Unit) admission does not take into account the evolution of sCr as a response to therapeutic interventions. Although this might not be problematic when "AKI" is used as a *diagnostic* classifier, it can have an influence on the *prognostic value* of the label "AKI".

The prognostic value of AKI, using a 0.3 mg/dl cut off value for sCr increase, as proposed by AKIN (Acute Kidney Injury Network), KDIGO (Kidney Disease Improving Global Outcomes) and ERBP (European Renal Best Practice) [5, 7, 8], has not previously been validated in a prospective cohort of exclusively sepsis patients. These patients are particularly prone to capillary leak and fluid accumulation which influences the distribution volume of sCr, potentially resulting in a delay in sCr increase due to dilution [13]. This might result in even smaller increases of serum creatinine being associated with mortality in sepsis.

We hypothesized that changing the way to calculate serum creatinine increase could impact the prognostic value of AKI. In this prospective cohort study, we evaluated the impact of using three different algorithms to calculate the sCr increase. One algorithm (ΔADM) took into account the evolution of sCr by comparing the sCr value 24 h after ICU admission with the ICU admission value. The other two algorithms (ΔHIS and ΔEST) were based on the *peak* sCr value over the same time span, compared to either a historical baseline value (ΔHIS) or an estimated baseline value (ΔEST). Additionally, we intended to explore the robustness of a 0.3 mg/dl sCr increase in sepsis, by comparing its predictive value to that of either smaller or larger sCr increases. We hypothesized that in sepsis, maybe even smaller serum creatinine increases would be associated with mortality because of dilution due to fluid accumulation.

## Methods

One hundred and ninety five consecutive adult patients (age ≥ 17 years) with sepsis admitted to the intensive care unit (ICU) of the Ghent University Hospital between 12/01/2010 and 27/03/2011 were included in this prospective cohort study. Sepsis, severe sepsis and septic shock were defined according to the American College of Chest Physicians/Society of Critical Care Medicine Consensus Conference guidelines [14]. Exclusion criteria were: 1) ICU stay less than 24 h or withdrawal of therapy, 2) no bladder catheter, 3) patients treated with chronic hemodialysis, 4) patients with RRT need due to AKI *upon* ICU admission, 5) Age < 17 years, 6) a history of organ transplantation, 7) obstructive AKI and 8) no central line or arterial catheter. Survival status was assessed at ICU, at 3 months, 1 year and 2 years by JV, either by checking hospital records or by telephone interview with the family practitioner.

Patients who developed sepsis *during* their ICU stay were not considered for inclusion.

The study was approved by the ethical committee of the Ghent University Hospital. Written informed consent was obtained from the patient or their next of kin.

Three algorithms were used to calculate the sCr increase: 1) “ΔHIS”, defined as the highest value within 24 h after ICU admission minus the value of a pre-admission historical baseline; 2) “ΔEST”, defined as the highest value 24 h after ICU admission minus an estimated baseline value obtained by solving the MDRD (Modification of Diet in Renal Disease) equation assuming a GFR (Glomerular Filtration Ratio) of 75 ml/min/1.73 m^2^, as suggested by ADQI [6] and 3) “ΔADM”, defined as the value at 24 h after ICU admission minus the value at ICU admission. We also used different values of sCr increase as threshold for AKI diagnosis (0.1, 0.2, 0.3, 0.4, and 0.5 mg/dl), to assess the robustness of the 0.3 mg/dl sCr increase criterion in sepsis.

Blood samples for measuring sCr were collected at the moment of study inclusion (D0T0), four hours later (D0T4), the next morning at 6 AM (D1) and daily at 6 AM for the next four days. during the first five days after ICU admission, centrifuged immediately and frozen at −80° for later batch analysis, using an Isotope Dilution Mass Spectroscopy traceable method (Roche Diagnostics®). Once patients were on dialysis, serum creatinine values within study protocol were no longer measured.

Severity of illness was assessed by the APACHE (Acute Physiology and Chronic Health Evaluation) II score during the first 24 h after admission, with or without the renal score.

The 24 h fluid balance was registered for all patients to account for bias due to fluid dilution.

**Fig. 1:**
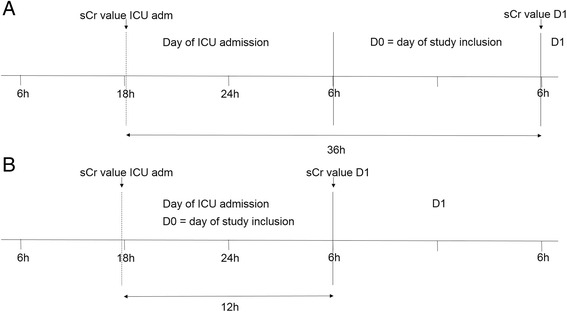
Illustration of the method used for study inclusion. **a**: Graphic presentation of the time interval between the sCr value at D1 and the ICU admission value in case of a patient being admitted just after 18 pm. **b**: Graphic presentation of the time interval between the sCr value at D1 and the ICU admission value in case of a patient being admitted just before 18 pm

All sepsis patients admitted to ICU between 6 AM and 18 PM were included on the day of ICU admission. Patients admitted to ICU after 18 PM were included the following day at 6 AM. For all patients, ‘Day 1’ (D1 = the day following the inclusion day) starts at 6 AM following the day of inclusion. (Fig. 1) Since the time interval between D1 and ICU admission was the same in each individual patient for the three algorithms, this issue is not likely to have influenced our results. The mean time interval also approximates 24 h (mean = 26 h). Additionally, if anything, having to deal with different time intervals between patients would have lowered our chances of demonstrating that including the evolution of serum creatinine in the AKI definition enhances the predictive performance of the label ‘AKI’, The latter because in those patients with a time interval less than 24 h, there might not have been sufficient time to evaluate the response to fluid resuscitation yet.

### Statistical analysis

Results are reported as medians and interquartile ranges (IQR) for continuous variables, unless otherwise specified. Discrete variables are reported as numbers and/or percentages. All statistical analyses were performed using SPSS® 19.

Chi square was used to asses a difference in prevalence in case of a dichotomous outcome. Results are reported as Odds ratio with 95 % confidence intervals (OR; 95 % CI). Student *t* test was used to compare the means of normally distributed continuous variables whereas non parametric tests were used to compare the medians of not normally distributed continuous variables.

Logistic regression (3 months) and Cox regression (1 and 2 year) were performed in forward and backward mode, presenting age, gender, presence of pre-existing renal insufficiency (eGFR <60 ml/min) (CKD (Chronic Kidney Disease)), APACHE II score, need for ventilation and 24 h fluid balance to the model as potential predictors for mortality. As additional parameter, AKI according to the different algorithms (ΔHIS, ΔEST and ΔADM) and with different cut-offs for sCr increase for each algorithm (0.1, 0.2, 0.3, 0.4 and 0.5 mg/dl sCr increase) were added to the model as a dummy code. Predictive performance was also assessed by comparing AUC ROC (Area Under the Curve of a Receiver Operating Characteristics Curve) analysis of the different models.

For the short term (3 months) mortality, we used a logistic regression model whereas for 1 and 2 years survival, we considered survival time as a continuous variable and used Cox Regression.

None of the patients was lost to follow-up. Two patients did not have a serum creatinine value available at D1 because they had died by then; as a consequence the ∆ADM algorithm could not be used in these patients (since it is based on the value at D1 minus the ICU admission value). Another 8 patients did not have a serum creatinine value available at D1 because they were already on RRT by that time. These patients all had a rise in serum creatinine > 0.3 mg/dl before reaching the end of the time interval (=the value at D1), so they were assumed to have AKI according to the ∆ADM algorithm although no actual value at D1 was available to calculate the serum creatinine increase. This paper complies with the STROBE criteria (see Additional file 1).

## Results

### Descriptive patient data

In this study, 195 consecutive patients with sepsis, severe sepsis and septic shock, were included. During the study period, 253 patients were considered for inclusion of whom 58 were excluded (18 for not having a bladder catheter, 13 because of RRT need upon ICU admission, 10 with a history of organ transplantation, 7 because of the decision to withdraw therapy, 5 for being treated with chronic dialysis, 3 who had ICU stay < 24 h, 1 with obstructive AKI and 1 who did not have an arterial or central venous line). Nine (4.6 %), 63 (32.3 %) and 123 (63.1 %) had sepsis, severe sepsis and septic shock, respectively. Overall mortality rates at the ICU, three months, 1 year and 2 years were 23.1 %, 31.3 %, 43.6 % and 50.3 %, respectively. Of the patients who needed RRT during their ICU stay (*n* = 27(13.8 %)), cumulative mortality rate during ICU stay was 55.6 % (OR 5.75(2.44-13.53). Eighty-three percent of patients who were treated with RRT and survived ICU (*n* = 10/12), survived up to two years. One patient treated with RRT died at three months and another died at year 1.

Demographics and relevant data for AKI vs no AKI according to the three different definitions (ΔADM > 0.3 mg/dl, ΔHIS > 0.3 mg/dl and ΔEST > 0.3 mg/dl) are presented in Table 1.

**Table 1:** Demographics in AKI vs no-AKI according to different definitions

AKI definition	ΔADM > 0.3			ΔHIS > 0.3			ΔEST > 0.3		
AKI/noAKI (n/%)	AKI (27/13.8)	no AKI (166/85.1)	p value	AKI(98/50.3)	no AKI (97/49.7)	p value	AKI (89/45.6)	no AKI (106/54.4)	p value
%male	66.7	61.4	0.6	67.3	57.7	0.17	65.2	60.4	0.49
Age(y)(mean/sd)	66.2(10.7)	60.7(15.6)	0.08	62.6(14.1)	60.2(15.8)	0.28	65.1(13.5)	58.2(15.5)	0.001
APACHE II	27(9)	22(9)	0.003	25(8)	19(9)	<0.001	25(8)	20(9)	<0.001
Ventilation(n/%)	25/92.6	80/48.2	<0.001	59/60.2	47/48.5	0.10	55/61.8	51/48.1	0.06
CKD(n/%)	7/25.9	23/13.9	0.11	18/18.4	12/12.4	0.25	25/28.1	5/4.7	<0.001
ICU admission serum creatinine (mg/dl)	1.29(0.66)	1.05(0.89)	0.01	1.67(1.25)	0.79(0.40)	<0.001	1.76(1.24)	0.80(0.39)	<0.001
Historical baseline creatinine (mg/dl)	0.98(0.41)	0.83(0.32)	0.18	0.88(0.37)	0.82(0.34)	0.52	0.98(0.35)	0.79(0.32)	0.001
RRT need(n/%)	14/51.9	13/7.8	<0.001	23/23.5	4/4.1	<0.001	24/27	3/2.8	<0.001
ICU LOS(d) in ICU survivors	15(22)	6(8)	0.009	6(9)	5(10)	0.34	7(9)	6(9)	0.24
ICU Mort(n/%)	12/44.4	31/18.7	0.003	25/25.5	20/20.6	0.42	22/24.7	23/21.7	0.62
Mort at three months(n/%)	13/48.1	46/27.7	0.03	32/32.7	29/39.9	0.68	30/33.7	31/29.2	0.50
Mort at 1 year(n/%)	17/63	66/39.8	0.024	42/42.9	43/44.3	0.84	41/46.1	44/41.5	0.52
Mort at 2 years(n/%)	17/63	79/47.6	0.14	47/48	51/52.6	0.52	46/48.3	51/51.9	0.62

*AKI* Acute kidney injury, ΔADM > 0.3 = Serum creatinine increase > 0.3 mg/dl based on the difference between the value 24 h after admission and ICU admission, ΔHIS > 0.3 = Serum creatinine increase > 0.3 mg/dl based on the difference between the highest value during the first 24 h after ICU admission and a historical baseline value, ΔEST > 0.3 = Serum creatinine increase > 0.3 mg/dl based on the difference between the highest value during the first 24 h after ICU admission and an estimated baseline value, *APACHE II* Acute physiology and chronic health evaluation II score, *CKD* Chronic kidney disease, *RRT* Renal replacement therapy, *ICU LOS* Intensive care unit length of stay, *Mort* Mortality

The prevalence of AKI varied according to the definition used. Based on ΔADM > 0.3 mg/dl vs ΔHIS > 0.3 mg/dl and ΔEST > 0.3 mg/dl, 27(13.8 %) vs 98(50.3 %) and 89(45.6) patients were labelled as having AKI (*p* < 0.001). (Table 1).

**Fig. 2:**
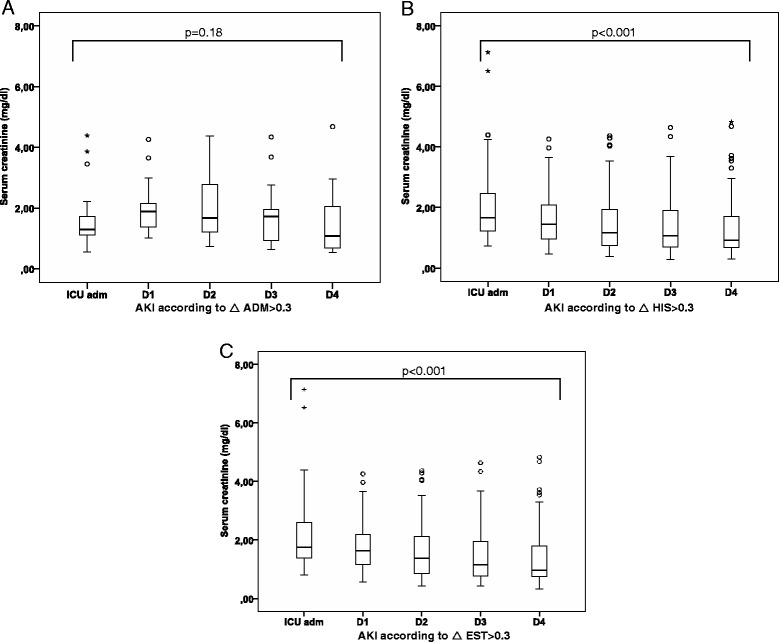
Evolution of serum creatinine over 4 days in patients with Acute Kidney Injury (AKI), according to different algorithms. As opposed to Acute Kidney Injury based on ΔADM > 0.3 mg/dl (**a**), there is a steady decrease in serum creatinine after admission over the following four days when AKI is defined according to ΔHIS > 0.3 mg/dl (**b**) or ΔEST > 0.3 mg/dl (**c**)

Patients classified in the AKI vs no AKI group according to ΔADM > 0.3 mg/dl had a greater severity of illness as demonstrated by a higher APACHE II score (*p* = 0.003), a higher need for invasive ventilation (*p* < 0.001) and a longer ICU stay (*p* = 0.009). When ΔHIS or ΔEST were used, there was a difference in APACHE II score in AKI vs no AKI but not in need for invasive ventilation or length of ICU stay (Table 1). Based on ΔHIS or ΔEST, there is a steady decrease in sCr vs the admission value in the AKI group over the following four days, as opposed to AKI according to ΔADM (Fig. 2a, b and c).

### Influence on prognostic value of different algorithms to calculate a serum creatinine increase of 0.3 mg/dl

#### Entire study cohort

**Fig. 3:**
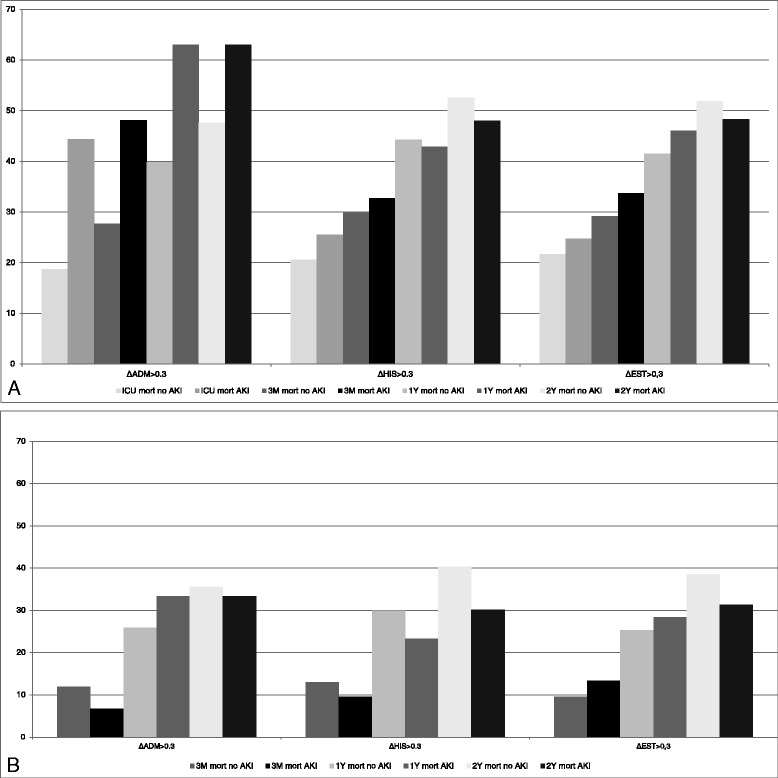
Mortality rates (%) in Acute Kidney Injury (AKI) vs no Acute Kidney Injury according to different algorithms, in the entire cohort and in ICU survivors separately. **a**: Mortality rates at ICU, 3 months, 1 year and 2 years in the entire cohort in AKI vs no AKI, either based on ΔADM > 0.3 mg/dl, ΔHIS > 0.3 mg/dl or ΔEST > 0.3 mg/dl. Only based on ΔADM > 0.3 mg/dl, there is a higher mortality at ICU, 3 months and 1 year in AKI vs no AKI. At year 2, there is no significant different in mortality between AKI and no AKI with either of the algorithms. **b**: Mortality rates at 3 months, 1 year and 2 years in ICU survivors in AKI vs no AKI, either based on ΔADM > 0.3 mg/dl, ΔHIS > 0.3 mg/dl or ΔEST > 0.3 mg/dl. There is no significant difference in mortality between AKI and no AKI at the three time points, independent of the algorithm used

In patients with AKI vs no AKI defined according to ΔADM > 0.3 mg/dl, ICU mortality, and three months, 1 year and 2 years mortality rates were 44.4 %, 48.1 %,63 % and 63 % vs 18.7 %, 27.7 %, 39.8 % and 47.6 %, respectively (OR 3.48(1.48-8.18), 2.42(1.06-5.54), 2.58(1.11-5.97) and 1.87(0.81-4.33)) (Table 1 and Fig. 3a).

When AKI was defined according to ΔHIS > 0.3 mg/dl or ΔEST > 0.3 mg/dl, mortality rates were not different between AKI and no-AKI respectively at any of the time points (Table 1 and Fig. 3a).

#### ICU survivors

In ICU survivors (*n* = 150), mortality rates at three months, 1 year and 2 years in AKI vs no-AKI according to ΔADM > 0.3 mg/dl were 6.7 %, 33.3 % and 33.3 % vs 11.9 %, 25.9 % and 35.6 % respectively (OR 0.53(0.07-4.32), 1.43(0.46-4.47) and 0.91(0.29-2.81) respectively). Based on ΔHIS > 0.3 mg/dl, mortality rates were 9.6 %, 23.3 % and 30.1 % vs 13 %, 29.9 % and 40.3 % respectively (OR 0.71(0.26-1.98), 0.71(0.34-1.48) and 0.64(0.33-1.26)). Based on ΔEST > 0.3 mg/dl these were 13.4 %, 28.4 % and 31.3 % vs 9.6 %, 25.3 % and 38.6 % in AKI vs no AKI respectively (OR 1.46(0.53-4.00), 1.17(0.57-2.42) and 0.73(0.37-1.44)) (Fig. 3b).

Thus with none of the above definitions (ΔADM > 0.3 mg/dl, ΔHIS > 0.3 mg/dl or ΔEST > 0.3 mg/dl) there was a significant difference in mortality rates between AKI and no AKI in septic patients who survived their ICU stay, either at three months, 1 year and 2 years.

Also other variants of calculating ΔsCr were assessed, which confirmed that in sepsis patients only the evolution of sCr as compared to admission sCr was prognostic for mortality (see Additional files 2, 3 and 4).

### Prognostic value for mortality of different cut-off values for serum creatinine increase

**Fig. 4:**
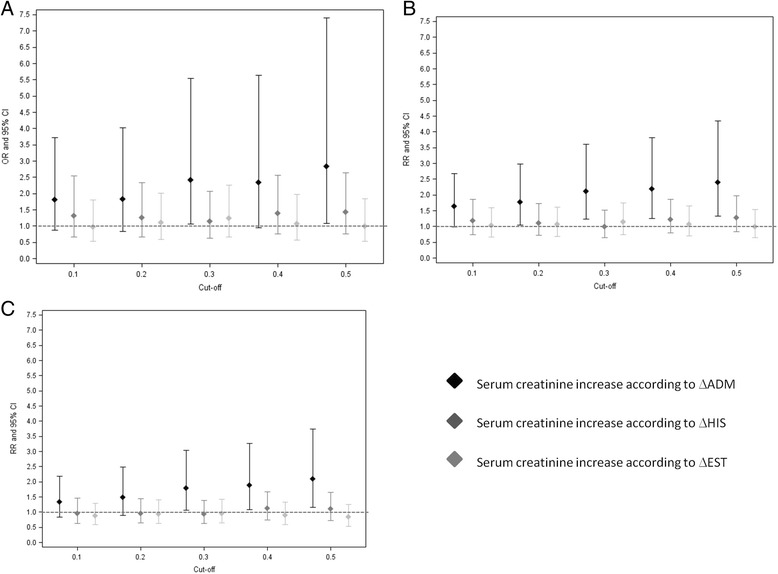
Odds ratio of incremental cut-off values for serum creatinine increase and mortality in the entire cohort. A 0.3 mg/dl increase in serum creatinine is the lowest robust cut-off value associated with 3 months mortality (**a**), but only if this increase is based on ΔADM > 0.3 mg/dl (OR 2.42(1.06-5.54). At year 1 (**b**) and year 2 (**c**), a serum creatinine increase of 0.3 mg/dl is also the lowest robust cut-off value associated with mortality but again only if this increase is based on ΔADM > 0.3 mg/dl (RR 2.11(1.24-3.6) and RR 1.79(1.06-3.03) at year 1 and year 2 respectively)

Using the different algorithms to calculate increase of sCr (ΔADM, ΔHIS, ΔEST) and different cut-off levels for that increase (0.1 mg/dl to 0.5 mg/dl with increments of 0.1 mg/dl), we found that in univariate analysis an increase in sCr of 0.3 mg/dl was the lowest robust cut-off value that was still associated with mortality at three months in the entire cohort (OR 2.42, 95 % CI 1.06-5.54), but only if the difference in increase of sCr was based on ΔADM (Fig. 4a). At year 1 and 2 an increase in sCr of 0.3 mg/dl was also the lowest robust cut-off for prediction of mortality in the entire cohort, again only when the definition is based on ΔADM (RR 2.11(1.24-3.6) and RR 1.79(1.06-3.03) for mortality at 1 year and 2 years respectively) (Fig. 4b and c). In multivariate analysis adjusting for the 24 h fluid balance, a 0.3 mg/dl increase in sCr remained the lowest robust value that was associated with mortality.

### Multivariate analysis for prediction of mortality

In a logistic regression model adjusted, besides fluid balance, for age and gender, APACHE II score (OR per point: 1.07, 95 % CI 1.02-1.13) and Need for Ventilation (OR 3.29, 95 % CI 1.55-6.98) but not AKI according to ΔADM, were independent predictors for mortality at three months (Table 2). Comparable results were obtained when the other definitions of AKI were used (Table 2). Using the APACHE II score without the renal score did not change these findings.

**Table 2:** Multivariate logistic regression for prediction of mortality at three months including different AKI definitions in the model

	Exp(B)	95 % CI	*p* value
*ΔADM > 0.3*	1.36	0.53-3.51	0.53
Age	1.01	0.99-1.04	0.28
Gender	0.75	0.37-1.47	0.39
APACHE II	1.07	1.02-1.13	0.009
Need for Ventilation	3.29	1.55-6.98	0.002
Fluid balance over the first 24 h after ICU admission	0.89	0.76-1.05	0.17
*ΔHIS > 0.3*	0.64	0.31-1.32	0.23
Age	1.01	0.99-1.04	0.35
Gender	0.68	0.34-1.35	0.27
APACHE II	1.09	1.03-1.15	0.002
Need for Ventilation	3.32	1.60-6.89	0.001
Fluid balance over the first 24 h after ICU admission	0.92	0.79-1.07	0.28
*ΔEST > 0.3*	0.66	0.32-1.36	0.26
Age	1.01	0.99-1.04	0.26
Gender	0.69	0.35-1.38	0.29
APACHE II	1.09	1.03-1.15	0.002
Need for Ventilation	3.32	1.60-6.87	0.001
Fluid balance over the first 24 h after ICU admission	0.92	0.78-1.07	0.27

ΔADM > 0.3 = Serum creatinine increase > 0.3 mg/dl based on the difference between the value 24 h after admission and ICU admission, *APACHE II* Acute physiology and chronic health evaluation II score, *ICU* Intensive care unit, ΔHIS > 0.3 = Serum creatinine increase > 0.3 mg/dl based on the difference between the highest value during the first 24 h after ICU admission and a historical baseline value, ΔEST > 0.3 = Serum creatinine increase > 0.3 mg/dl based on the difference between the highest value during the first 24 h after ICU admission and an estimated baseline value

AUC ROC analysis confirmed that ΔADM > 0.3 mg/dl did not add discriminatory value above APACHE II score and Need for Ventilation for prognostication of mortality at 3 months (AUC 0.71 without ΔADM > 0.3 mg/dl and AUC 0.71 with ΔADM > 0.3 mg/dl).

In a Cox regression multivariate model applied to ICU survivors, only age was an independent predictor for mortality both at 1 year (RR = 1.04, 95 % CI: 1.01-1.07) and 2 years (RR = 1.02, 95 % CI: 1.002-1.045) (Table 3). This suggests that longer term mortality in sepsis patients is more influenced by underlying comorbidity such as older age than by AKI or other parameters of acute severity of illness.

**Table 3:** Multivariate cox regression analysis for prediction of mortality at 1 year and 2 years in ICU survivors

1 year mortality in ICU survivors	Exp(B)	95 % CI	*p* value
Age	1.04	1.01-1.07	0.004
Creatinine day 1	0.69	0.38-1.26	0.23
CKD	1.66	0.70-3.94	0.26
ΔADM > 0.3	1.60	0.48-5.31	0.44
Need for Ventilation	1.16	0.59-2.29	0.67
Gender	0.65	0.32-1.31	0.23
APACHE II	1.01	0.96-1.07	0.59
Fluid balance over the first 24 h after ICU admission	0.91	0.76-1.09	0.28
2 years mortality in ICU survivors			
Age	1.02	1.002-1.045	0.03
Creatinine day 1	0.48	0.26-0.86	0.01
CKD	2.06	0.94-4.48	0.07
ΔADM > 0.3	1.35	0.43-4.25	0.61
Need for Ventilation	1.44	0.79-2.60	0.23
Gender	0.68	0.37-1.24	0.20
APACHE II	1.04	0.99-1.08	0.10
Fluid balance over the first 24 h after ICU admission	0.91	0.78-1.06	0.21

*ICU* Intensive care unit, *CKD* Chronic kidney disease, ΔADM > 0.3 = Serum creatinine increase > 0.3 mg/dl based on the difference between the value 24 h after admission and ICU admission, *APACHE II* Acute physiology and chronic health evaluation II score

## Discussion

This study demonstrates that the predictive value of an increase in serum creatinine of 0.3 mg/dl, on mortality in sepsis, depends on how this sCr increase is calculated. Only if the evolution of sCr after ICU admission, rather than a peak sCr value over the same time span, is taken into account, an association with mortality is found. An increase in sCr of 0.3 mg/dl according to the ICU admission value is the lowest robust value still associated with mortality, confirming previous data in the cardiac surgery setting. This 0.3 mg/dl cut off remained robust even after adjustment for 24 h fluid balance. However, after adjusting for severity of illness, a sCr increase of 0.3 mg/dl was no longer associated with mortality.

Although the definition of AKI has become progressively more uniform since the introduction of RIFLE [6], uncertainty and debate on the methodology to calculate the sCr increase remains, and different interpretations are still appearing in the literature [9–12].

Most guidelines advocate to use the difference between the highest sCr value over a certain time span, and a pre admission historical baseline value [15, 16] or an estimated sCr value by solving the MDRD formula assuming a GFR of 75 ml/min/1,73 m^2^ [17–20]. It has been demonstrated that the use of surrogate baseline sCr values can lead to misclassification by either under- or overestimation of AKI [15]. Siew et al. investigated the impact of using different surrogate baseline values in a large cohort of 4863 adults, on AKI diagnosis and outcome [16]. As in our cohort, they found that the incidence of AKI decreased by using the ICU admission sCr value as baseline versus a pre admission historical sCr value. They also demonstrated that mortality rates were different according to which surrogate value was used [16]. As in our study, mortality rates were highest when AKI was defined according to the admission value. However, Siew et al. used a cohort containing mainly non-critically ill patients (only 19 % of patients were admitted to ICU) and only in-hospital and 60 days mortality were evaluated, without adjustment for severity of illness [16].

The strategy of using the peak sCr value over a certain time span after admission does not allow to study the impact of the evolution of sCr after starting therapeutic interventions such as fluid resuscitation. In our cohort, AKI was associated with mortality, but only if based on the difference between the value 24 h after admission and the value at ICU admission and not if based on the peak value over the same time span compared to a historical or estimated baseline value. These findings suggest that the evolution of sCr in the first 24 h after ICU admission, and thus the potential response to fluid resuscitation, is most predictive for outcome which is in line with previous observations [21]. By taking into account the evolution of serum creatinine after ICU admission, those patients who experience a decrease in serum creatinine under fluid resuscitation are no longer classified as having AKI which translates in a lower number of AKI cases compared to using the peak serum creatinine value over the same time period. It allows us to differentiate patients who are generally sicker and have more severe AKI and hence a worse outcome, from those with less severe AKI, responding to fluid resuscitation.

It is generally accepted that AKI is associated with increased mortality [22–24]. Bagshaw et al. performed a retrospective interrogation of prospectively included patients with sepsis, septic AKI and non-septic AKI. Sepsis, septic AKI and non-septic AKI were all found to be significantly associated with poor outcome. However, authors only looked at short term mortality (28 days and hospital mortality) and no data on RRT need were available, so it is unclear whether the effect on mortality was mainly driven by RRT need [23].

Despite this generally accepted association between AKI and mortality, the topic is still also a matter of debate, even in the non-critically ill. In a population based study of AKI, no association between AKI and outcome was found [25]. A higher risk for chronic dialysis need, but not mortality, was found in a large cohort of ICU survivors [26]. However, several reports indicated that the *duration* of AKI is highly predictive of mortality [27], an aspect that was not evaluated in the current study. This might explain why in our cohort the diagnosis of AKI was no longer independently associated with mortality after adjustment for severity of illness. Our data indicate that the response to treatment in the first 24 h after ICU admission also influences prognosis, probably by unveiling those who positively respond to volume resuscitation. Several older and more recent studies also demonstrated that the most important predictors for mortality were already present at admission to the ICU and included advanced age, the presence of infection, a past history of chronic diseases and the presence of other failing organs [28–32].

Although recent literature shows that even small increases in sCr are independently associated with mortality, these results are mainly obtained in cohorts of cardiac surgery patients, after coronarography and after myocardial infarction [1–4, 33–35]., In cardiac surgery, the relation between small increases in sCr and mortality might be amplified by the observation that in patients without AKI, sCr levels are expected to decrease in the first 24 h after surgery because of fluid loading perioperatively, diluting the sCr value [36]. The association between small increases in sCr and poor outcome cannot necessarily be extrapolated to other conditions, such as sepsis. Sepsis patients are particularly vulnerable to capillary leak and fluid accumulation. Thus theoretically even smaller increases of sCr could be associated with mortality because of dilution of sCr, and a delay in diagnosis [13]. However, according to the results of the current study, even after adjustment for 24 h fluid balance, a 0.3 mg/dl increase in sCr 24 h after ICU admission compared to the ICU admission value, seems to be the lowest robust threshold for increased risk in sepsis patients.

The strong points of this prospective study are the availability of longer term outcomes and the detailed patient information.

Our study is the first to consider that the evolution of sCr after start of therapy, rather than an absolute highest value over a certain time span is important with regard to outcome.

A limitation of this study is its observational nature and the fact that it is a single centre study describing a relatively small cohort of patients which enhances the chance of a type 2 error. We acknowledge that our results need to be validated in a larger cohort and are not necessarily generalizable to sepsis cohorts including patients with different severity of illness. However, the finding that taking into account the evolution of serum creatinine demonstrates a better association with mortality compared to only relying on a peak serum creatinine value over a certain time span for AKI diagnosis, does make sense from a pathophysiological point of view. Taking into account the evolution of serum creatinine allows for the identification of those who have more sever AKI, are more ill and consequently have a worse outcome.

By capturing AKI during the first 24 h after ICU admission we could incorporate the early response to fluid resuscitation in the ∆ADM algorithm. Although by doing so we missed the AKI cases occurring after 24 h this is not likely to change our general message that AKI classification criteria should incorporate the evolution of sCr in response to fluid resuscitation. Also when we used a 48 h interval, our findings did not change (see Additional files 2, 3 and 4).

We did not include the urinary output as a prognostic criterion in this study. We demonstrated before that taking into account urinary output improved the diagnostic performance of RIFLE [37]. In several other studies, it was demonstrated that urinary output is associated with mortality in the critically ill [38, 39]. In the current study, however, we wanted to evaluate the independent impact a change in the sCr criterion, as this is the most widely used criterion for prognostic modelling, especially in large (administrative) database cohorts [35, 40, 41].

Mortality rates in our cohort are low compared to what was reported in a recent study [42]. However, in general, mortality rates in sepsis have been reported to decline [42–45]. Mortality rates in our cohort of patients with sepsis might also be lower than in previous reports because we have a very good system in place in our ICU to alert physicians of pending AKI. Most of these alerts are induced by reduced urinary output [46]. It has been demonstrated before that AKI defined by oliguria has a better prognosis as AKI defined by the creatinine criterion [24].

## Conclusion

The prognostic value of a 0.3 mg/dl increase in sCr, on mortality in sepsis, depends on the algorithm to calculate this sCr increase. Only if the evolution of serum creatinine over the first 24 h after ICU admission is taken into account, an association with mortality is found. This 0.3 mg/dl increase criterion remains robust, even after adjusting for 24 fluid balance. After adjustment for severity of illness, a sCr increase of 0.3 mg/dl is no longer associated with mortality in sepsis.

## Additional files

Additional file 1:
**STROBE checklist.**
Additional file 2:
**Odds ratio of incremental cut-off values for serum creatinine increase and 3 months mortality in the entire cohort.**
Additional file 3:
**Relative Risk of incremental cut-off values for serum creatinine increase and 1 year mortality in the entire cohort.**
Additional file 4:
**Relative Risk of incremental cut-off values for serum creatinine increase and 2 years mortality in the entire cohort.**

## Abbreviations

∆ADM: The difference between the serum creatinine value 24 h after ICU admission and the ICU admission value
ADQI: Acute dialysis quality initiative
AKI: Acute kidney injury
AKIN: Acute kidney injury network
APACHE II score: Acute physiology and chronic health evaluation II score
AUC ROC: Area under the curve of a receiver operating characteristics curve
CRP: C-reactive protein
CKD: Chronic kidney disease
(e)GFR: (estimated) glomerular filtration ratio
ERBP: European renal best practice
∆EST: The difference between the highest value serum creatinine value over the first 24 h after ICU admission and an estimated baseline value
∆HIS: The difference between the highest serum creatinine value over the first 24 h after ICI admission and a historical baseline value
ICU: Intensive care unit
IQR: Interquartile range
KDIGO: Kidney disease improving global outcome
LOS: Length of stay
MDRD: Modification of diet in renal disease
OR: Odds ratio
RIFLE: Risk, injury, failure, loss of kidney function and end-stage renal disease
RR: Relative risk
RRT: Renal replacement therapy
sCr: serum creatinine
