# Supplementary material for: Prognostic robustness of serum creatinine based AKI definitions in patients with sepsis: a prospective cohort study
Source: BMC Nephrol. 2015 Jul 22;16:112. doi: 10.1186/s12882-015-0107-4 (PMC4511260; doi:10.1186/s12882-015-0107-4)
Supplement: Additional file 1: — STROBE checklist. [file 12882_2015_107_MOESM1_ESM.doc]

STROBE Statement—Checklist of items that should be included in reports of ***cohort studies***

|  | Item No | Recommendation |
| --- | --- | --- |
| **Title and abstract** | 1 | *(*a) Indicate the study’s design with a commonly used term in the title or the abstract  This has been done in the title (“…: a prospective cohort study” and in the methods’ section of the abstract (“We prospectively included…”). |
|  |  | (b) Provide in the abstract an informative and balanced summary of what was done and what was found  The abstract provides an informative and balanced summary of what was done and what was found. Our hypothesis was that changing the way serum creatinine increase was calculated, would influence the association between AKI and mortality. What we found was that only if the evolution of serum creatinine was taken into account when calculating this serum creatinine increase, there was an association with mortality. (p2) |
| Introduction | | |
| Background/rationale | 2 | Explain the scientific background and rationale for the investigation being reported  This has been done (page 5). Despite the widely accepted RIFLE,AKIN and KDIGO criteria for diagnosing AKI, there is still lack of clarity on how to calculate the serum creatinine increase and different interpretations of the same definitions are being used. The creatinine criterion for AKI diagnosis mostly relates to a serum creatinine increase compared to a historical baseline value (except for AKIN which uses the ICU admission value as a baseline value). However, this historical baseline value if often unknown and using surrogate baseline values might lead to either under- or overestimation of AKI. Recent literature also demonstrated that even small serum creatinine increases (≤0.3 mg/dl) are associated with mortality; however this has not been tested in exclusively sepsis patients. |
| Objectives | 3 | State specific objectives, including any prespecified hypotheses  In this study we used different baseline values to calculate serum creatinine increase and in one of the algorithms used, we also took into account the fact whether creatinine was increasing or decreasing instead of only using the peak creatinine value over a certain time period to diagnose AKI. We tested different cut-off values for serum creatinine increase (0.1 to 0.5 mg/dl with incremental increases of 0.1 mg/dl). We hypothesized that the way we calculated the serum creatinine increase might influence the association between AKI and mortality. We also hypothesized that maybe smaller increases in serum creatinine than 0.3 mg/dl might be associated with mortality because dilution due to capillary leak with fluid accumulation might cause a more important delay in serum creatinine increase. (p5-6 last paragraph) |
| Methods | | |
| Study design | 4 | Present key elements of study design early in the paper  This has been done in the first paragraph of page 7. |
| Setting | 5 | Describe the setting, locations, and relevant dates, including periods of recruitment, exposure, follow-up, and data collection  All these data have been included in the methods’ section (page 7). All patients admitted to ICU at the Ghent University Hospital between 12/01/2010 and 27/03/2011 with sepsis, severe sepsis or septic shock were considered for inclusion. All data were collected by JV. The study was approved by the ethical committee of the Ghent University Hospital. Written informed consent was obtained from the patient or the next of kin. |
| Participants | 6 | (a) Give the eligibility criteria, and the sources and methods of selection of participants. Describe methods of follow-up  These data are provided on page 7.  Patients were eligible if they were admitted with sepsis, severe sepsis or septic shock except if the following exclusion criteria were present: 1) ICU stay less than 24 hours or withdrawal of therapy, 2) no bladder catheter, 3) patients treated with chronic hemodialysis, 4) patients with RRT need due to AKI upon ICU admission, 5) Age < 17 years, 6) a history of organ transplantation, 7) obstructive AKI and 8) no central line or arterial catheter. JV daily screened for new patients at ICU between 6AM AND 18PM. Patients admitted after 18PM were included the following day. For all patients, ‘Day 1’ (D1=the day following the inclusion day) started at 6 AM following the day of inclusion. For each individual patient the time interval that is used to calculate the serum creatinine increase according to the different algorithms (either ∆ADM, ∆HIS or ∆EST) is the same, however, in between patients this time interval might differ. The mean time interval between D1 and ICU admission in our cohort was 26 hours, which approximates 24h. |
|  |  | (*b*) For matched studies, give matching criteria and number of exposed and unexposed |
| Variables | 7 | Clearly define all outcomes, exposures, predictors, potential confounders, and effect modifiers. Give diagnostic criteria, if applicable  Sepsis, severe sepsis and septic shock were defined according to the ACCP/SCCM criteria. AKI was based on serum creatinine increase only, because we wanted to evaluate the robustness of creatinine based AKI definitions in sepsis. We previously had shown that oliguria is important to avoid missing AKI cases but in this study we were interested in the impact of changing the way to calculate serum creatinine increase on the predictive performance of the label ‘AKI’. We used three different algorithms to define AKI and also used different cut off values for serum creatinine increase within each algorithm as explained on page 7- 8. Because sepsis can have an influence on outcome well beyond the commonly accepted ’28 days’ and because we were also interested in median term outcome, we used 90 days mortality, 1 year and 2 years mortality rates as outcome measures. We evaluated the impact of AKI according to different definitions on outcome after adjustment of severity of illness (APACHE II score (with or without the renal score) and need for ventilation) for 3 months mortality and also for comorbidities (age, CKD) for 1 year and 2 years mortality because these variables might confound the relationship between AKI and mortality. |
| Data sources/ measurement | 8* | For each variable of interest, give sources of data and details of methods of assessment (measurement). Describe comparability of assessment methods if there is more than one group  This information can be found on page 7-8.  Blood samples for measuring sCr were collected at the moment of study inclusion (D0T0), four hours later (D0T4), the next morning at 6AM (D1) and daily at 6AM for the next four days. Additional creatinine values obtained by samples that were performed outside the study protocol (at the discretion of the intensivist) were also taken into account. Survival status was ascertained by checking hospital records or by telephone interview with the family practitioner. Blood samples were drawn by JV and analysed in the hospital lab and data on survival status were collected by JV. |
| Bias | 9 | Describe any efforts to address potential sources of bias  We estimated that confounding bias could arise by severity of illness (since patients with a higher severity of illness are both more likely to develop AKI and to die) and by fluid dilution. This is explained on page 8.  Possible bias has also been stressed in the study limitations on page 18. |
| Study size | 10 | Explain how the study size was arrived at  No power calculation was done in this observational study |
| Quantitative variables | 11 | Explain how quantitative variables were handled in the analyses. If applicable, describe which groupings were chosen and why  This is explained on page 8 in the paragraph about the statistical analysis  Quantitative variables were compared between predefined groups of AKI vs no AKI according to the different algorithms |
| Statistical methods | 12 | (a) Describe all statistical methods, including those used to control for confounding  This is explained on page 8 in the paragraph about the statistical analysis |
|  |  | (*b*) Describe any methods used to examine subgroups and interactions  Post hoc interaction terms for AKI and CKD and AKI and APACHE II score were incorporated in the model and did not change findings. |
|  |  | (*c*) Explain how missing data were addressed  Two patients did not have a serum creatinine value available at D1 because they had died by then; as a consequence the ∆ADM algorithm could not be used in these patients (since it is based on the value at D1 minus the ICU admission value). Another 8 patients did not have a serum creatinine value available at D1 because they were already on RRT by that time (but not at the moment of ICU admission= exclusion criterion). These patients all had a rise in serum creatinine > 0.3 mg/dl befor reaching the end of the time interval (=the value at D1), so it they were assumed to have AKI according to the ∆ADM algorithm although no value at D1 was available to calculate the serum creatinine increase. No patients were lost to follow-up. |
|  |  | (*d*) If applicable, explain how loss to follow-up was addressed  None of the patients was lost to follow-up. |
|  |  | (*e*) Describe any sensitivity analyses  We performed a sensitivity analysis testing different cut-off levels for serum creatinine increase (0.1 mg/dl till 0.5 mg/dl, using incremental increases of 0.1 mg/dl) within each algorithm (either ∆ADM, ∆HIS or ∆EST). No other predefined sensitivity analysis was done. |
| Results | | |
| Participants | 13* | (a) Report numbers of individuals at each stage of study—eg numbers potentially eligible, examined for eligibility, confirmed eligible, included in the study, completing follow-up, and analysed  These data are provided in the results’ section on page 11.  During the study period, 253 patients were considered for inclusion of whom 58 were excluded (18 for not having a bladder catheter, 13 because of RRT need upon ICU admission, 10 with a history of organ transplantation, 7 because of the decision to withdraw therapy, 5 for being treated with chronic dialysis, 3 who had an ICU stay < 24h, 1 with obstructive AKI and 1 who did not have an arterial or central venous line). For comparison of demographic data between AKI and no AKI for the different algoritms: see Table 1. |
|  |  | (b) Give reasons for non-participation at each stage  There were no cases of non-participation after study inclusion. |
|  |  | (c) Consider use of a flow diagram  A flow diagram was, in our opinion, not of additional value |
| Descriptive data | 14* | (a) Give characteristics of study participants (eg demographic, clinical, social) and information on exposures and potential confounders |
|  |  | (b) Indicate number of participants with missing data for each variable of interest |
|  |  | (c) Summarise follow-up time (eg, average and total amount)  Answer a-c: For demographics of the patients included see Table 1. None of the patients were lost to follow-up (all patients were followed-up for 2 years except of course those who died before reaching this time point). Two patients did not have a serum creatinine value at D1 because they had died; as a consequence the ∆ADM algorithm could not be used in these patients (since it is based on the value at D1 minus the ICU admission value). Another 8 patients did not have a serum creatinine value available at D1 because they were already on RRT by that time (but not at the moment of ICU admission= exclusion criterion). These patients all had a rise in serum creatinine > 0.3 mg/dl before reaching the end of the time interval (=the value at D1), so they were assumed to have AKI according to the ∆ADM algorithm although no value at D1 was available to calculate the serum creatinine increase. |
| Outcome data | 15* | Report numbers of outcome events or summary measures over time  Results on outcome are reported on page 12-14 and in Table 1. |
| Main results | 16 | (*a*) Give unadjusted estimates and, if applicable, confounder-adjusted estimates and their precision (eg, 95% confidence interval). Make clear which confounders were adjusted for and why they were included |
|  |  | (*b*) Report category boundaries when continuous variables were categorized |
|  |  | (*c*) If relevant, consider translating estimates of relative risk into absolute risk for a meaningful time period  Answer a-c: Main results are provided in tables 1,2 and 3 and figure 4 |
| Other analyses | 17 | Report other analyses done—eg analyses of subgroups and interactions, and sensitivity analyses  Post hoc we included an interaction term for AKI-CKD and AKI-severity of illness to the model. Sensitivity analysis using different cut off values for serum creatinine increase was done. |
| Discussion | | |
| Key results | 18 | Summarise key results with reference to study objectives  Key results are summarized with reference to the study objectives in the first paragraph on page 15. |
| Limitations | 19 | Discuss limitations of the study, taking into account sources of potential bias or imprecision. Discuss both direction and magnitude of any potential bias  Limitations are discussed on page 18. |
| Interpretation | 20 | Give a cautious overall interpretation of results considering objectives, limitations, multiplicity of analyses, results from similar studies, and other relevant evidence  This information is provided on page 18. |
| Generalisability | 21 | Discuss the generalisability (external validity) of the study results  This is discussed in the final paragraph on page 18. |
| Other information | | |
| Funding | 22 | Give the source of funding and the role of the funders for the present study and, if applicable, for the original study on which the present article is based  JV was funded by the Klinisch Onderzoeks Fonds of the Ghent University Fund. The funders did not have any role in the study itself. |

*Give information separately for exposed and unexposed groups.

**Note:** An Explanation and Elaboration article discusses each checklist item and gives methodological background and published examples of transparent reporting. The STROBE checklist is best used in conjunction with this article (freely available on the Web sites of PLoS Medicine at http://www.plosmedicine.org/, Annals of Internal Medicine at http://www.annals.org/, and Epidemiology at http://www.epidem.com/). Information on the STROBE Initiative is available at http://www.strobe-statement.org.
