# Supplementary material for: Prognostic robustness of serum creatinine based AKI definitions in patients with sepsis: a prospective cohort study
Source: BMC Nephrol. 2015 Jul 22;16:112. doi: 10.1186/s12882-015-0107-4 (PMC4511260; doi:10.1186/s12882-015-0107-4)
Supplement: Additional file 2: — Odds ratio of incremental cut-off values for serum creatinine increase and 3 months mortality in the entire cohort. [file 12882_2015_107_MOESM2_ESM.doc]

Supplemental Table1: Odds ratio of incremental cut-off values for serum creatinine increase and 3 months mortality in the entire cohort

| Definition | Cut-off | OR | CI lower | CI higher | p value |
| --- | --- | --- | --- | --- | --- |
| ∆H48h_histBL | 0.1 | 1,75 | 0,86 | 3,56 | 0,13 |
|  | 0.2 | 1,49 | 0,77 | 2,89 | 0,24 |
|  | 0.3 | 1,27 | 0,69 | 2,33 | 0,45 |
|  | 0.4 | 1,6 | 0,87 | 2,95 | 0,13 |
|  | 0.5 | 1,53 | 0,83 | 2,81 | 0,18 |
| ∆H48h_ICUadm | 0.1 | 1,29 | 0,7 | 2,39 | 0,42 |
|  | 0.2 | 1,13 | 0,57 | 2,27 | 0,72 |
|  | 0.3 | 1,5 | 0,72 | 3,11 | 0,28 |
|  | 0.4 | 1,86 | 0,84 | 4,13 | 0,13 |
|  | 0.5 | 2,24 | 0,93 | 5,4 | 0,07 |
| ∆H48h_estBL | 0.1 | 0,98 | 0,53 | 1,84 | 0,96 |
|  | 0.2 | 1,19 | 0,65 | 2,18 | 0,58 |
|  | 0.3 | 1,36 | 0,74 | 2,5 | 0,32 |
|  | 0.4 | 1,11 | 0,6 | 2,04 | 0,75 |
|  | 0.5 | 1,03 | 0,56 | 1,91 | 0,93 |
| ∆H24h_ICUadm | 0.1 | 1,33 | 0,7 | 2,5 | 0,38 |
|  | 0.2 | 1,3 | 0,62 | 2,72 | 0,49 |
|  | 0.3 | 1,35 | 0,58 | 3,15 | 0,49 |
|  | 0.4 | 2,4 | 0,9 | 6,4 | 0,08 |
|  | 0.5 | 2,9 | 0,75 | 11,2 | 0,12 |
| ∆D1 _histBL | 0.1 | 1,8 | 0,98 | 3,32 | 0,059 |
|  | 0.2 | 1,62 | 0,88 | 3 | 0,12 |
|  | 0.3 | 1,86 | 0,998 | 3,46 | 0,051 |
|  | 0.4 | 1,82 | 0,96 | 3,44 | 0,065 |
|  | 0.5 | 1,73 | 0,9 | 3,32 | 0,1 |
| ∆D1 _estBL | 0.1 | 1,3 | 0,7 | 2,39 | 0,41 |
|  | 0.2 | 1,52 | 0,82 | 2,83 | 0,19 |
|  | 0.3 | 1,58 | 0,84 | 2,95 | 0,16 |
|  | 0.4 | 1,84 | 0,97 | 3,49 | 0,06 |
|  | 0.5 | 2,01 | 1,04 | 3,87 | 0,04 |

*∆H48h_histBL*= Serum creatinine increase based on the highest value during the first 48h after admission versus a historical baseline value, *∆H48h_ICUadm*=Serum creatinine increase based on the highest value during the first 48h after admission versus the ICU admission value, *∆H48h_estBL*=Serum creatinine increase based on the highest value during the first 48h after admission versus an estimated baseline value, *∆H24h_ICUadm*=Serum creatinine increase based on the highest value during the first 24h after admission versus the ICU admission value, *∆D1_histBL*=Serum creatinine increase based on the value on D1 (24h after admission) versus a historical baseline value, *∆D1_estBL*=Serum creatinine increase based on the value on D1 (24h after admission) versus an estimated baseline value.
