# Supplementary material for: Prognostic robustness of serum creatinine based AKI definitions in patients with sepsis: a prospective cohort study
Source: BMC Nephrol. 2015 Jul 22;16:112. doi: 10.1186/s12882-015-0107-4 (PMC4511260; doi:10.1186/s12882-015-0107-4)
Supplement: Additional file 3: — Relative Risk of incremental cut-off values for serum creatinine increase and 1 year mortality in the entire cohort. [file 12882_2015_107_MOESM3_ESM.doc]

Supplemental Table2: Relative Risk of incremental cut-off values for serum creatinine increase and 1 year mortality in the entire cohort

| Definition | Cut-off | RR | CI lower | CI higher | p value |
| --- | --- | --- | --- | --- | --- |
| ∆H48h_histBL | 0.1 | 1,34 | 0,82 | 2,19 | 0,25 |
|  | 0.2 | 1,33 | 0,84 | 2,13 | 0,23 |
|  | 0.3 | 1,08 | 0,7 | 1,65 | 0,73 |
|  | 0.4 | 1,35 | 0,88 | 2,06 | 0,17 |
|  | 0.5 | 1,36 | 0,89 | 2,08 | 0,16 |
| ∆H48h_ICUadm | 0.1 | 1,15 | 0,75 | 1,77 | 0,53 |
|  | 0.2 | 1,18 | 0,73 | 1,9 | 0,51 |
|  | 0.3 | 1,37 | 0,83 | 2,27 | 0,22 |
|  | 0.4 | 1,74 | 1,03 | 2,93 | 0,04 |
|  | 0.5 | 2,01 | 1,15 | 3,52 | 0,01 |
| ∆H48h_estBL | 0.1 | 1,06 | 0,68 | 1,65 | 0,8 |
|  | 0.2 | 1,14 | 0,75 | 1,75 | 0,54 |
|  | 0.3 | 1,27 | 0,83 | 1,94 | 0,28 |
|  | 0.4 | 1,13 | 0,74 | 1,74 | 0,57 |
|  | 0.5 | 1,05 | 0,68 | 1,61 | 0,83 |
| ∆H24h_ICUadm | 0.1 | 1,19 | 0,77 | 1,86 | 0,43 |
|  | 0.2 | 1,32 | 0,8 | 2,18 | 0,28 |
|  | 0.3 | 1,35 | 0,76 | 2,4 | 0,3 |
|  | 0.4 | 2,11 | 1,15 | 3,89 | 0,02 |
|  | 0.5 | 2,76 | 1,27 | 5,99 | 0,01 |
| ∆D1 _histBL | 0.1 | 1,47 | 0,96 | 2,25 | 0,08 |
|  | 0.2 | 1,4 | 0,91 | 2,15 | 0,12 |
|  | 0.3 | 1,52 | 0,99 | 2,33 | 0,06 |
|  | 0.4 | 1,6 | 1,03 | 2,48 | 0,04 |
|  | 0.5 | 1,54 | 0,99 | 2,42 | 0,06 |
| ∆D1 _estBL | 0.1 | 1,23 | 0,8 | 1,89 | 0,34 |
|  | 0.2 | 1,48 | 0,96 | 2,27 | 0,08 |
|  | 0.3 | 1,48 | 0,96 | 2,29 | 0,08 |
|  | 0.4 | 1,55 | 0,998 | 2,41 | 0,051 |
|  | 0.5 | 1,53 | 0,97 | 2,41 | 0,07 |

*∆H48h_histBL*= Serum creatinine increase based on the highest value during the first 48h after admission versus a historical baseline value, *∆H48h_ICUadm*=Serum creatinine increase based on the highest value during the first 48h after admission versus the ICU admission value, *∆H48h_estBL*=Serum creatinine increase based on the highest value during the first 48h after admission versus an estimated baseline value, *∆H24h_ICUadm*=Serum creatinine increase based on the highest value during the first 24h after admission versus the ICU admission value, *∆D1_histBL*=Serum creatinine increase based on the value on D1 (24h after admission) versus a historical baseline value, *∆D1_estBL*=Serum creatinine increase based on the value on D1 (24h after admission) versus an estimated baseline value.
