# Supplementary material for: Prognostic robustness of serum creatinine based AKI definitions in patients with sepsis: a prospective cohort study
Source: BMC Nephrol. 2015 Jul 22;16:112. doi: 10.1186/s12882-015-0107-4 (PMC4511260; doi:10.1186/s12882-015-0107-4)
Supplement: Additional file 4: — Relative Risk of incremental cut-off values for serum creatinine increase and 2 years mortality in the entire cohort. [file 12882_2015_107_MOESM4_ESM.doc]

Supplemental Table 3: Relative Risk of incremental cut-off values for serum creatinine increase and 2 years mortality in the entire cohort

| Definition | Cut-off | RR | CI lower | CI higher | p value |
| --- | --- | --- | --- | --- | --- |
| ∆H48h_histBL | 0.1 | 1,04 | 0,67 | 1,6 | 0,86 |
|  | 0.2 | 1,09 | 0,72 | 1,66 | 0,68 |
|  | 0.3 | 0,98 | 0,66 | 1,46 | 0,93 |
|  | 0.4 | 1,2 | 0,81 | 1,79 | 0,36 |
|  | 0.5 | 1,15 | 0,77 | 1,71 | 0,5 |
| ∆H48h_ICUadm | 0.1 | 1,09 | 0,73 | 1,64 | 0,67 |
|  | 0.2 | 1,09 | 0,69 | 1,71 | 0,72 |
|  | 0.3 | 1,14 | 0,7 | 1,87 | 0,59 |
|  | 0.4 | 1,49 | 0,89 | 2,48 | 0,13 |
|  | 0.5 | 1,75 | 1,01 | 3,03 | 0,05 |
| ∆H48h_estBL | 0.1 | 0,91 | 0,61 | 1,37 | 0,65 |
|  | 0.2 | 1 | 0,67 | 1,49 | 1 |
|  | 0.3 | 1,05 | 0,71 | 1,56 | 0,82 |
|  | 0.4 | 0,92 | 0,61 | 1,38 | 0,69 |
|  | 0.5 | 0,86 | 0,57 | 1,3 | 0,48 |
| ∆H24h_ICUadm | 0.1 | 1,17 | 0,77 | 1,76 | 0,47 |
|  | 0.2 | 1,25 | 0,78 | 2,01 | 0,36 |
|  | 0.3 | 1,15 | 0,65 | 2,02 | 0,64 |
|  | 0.4 | 1,85 | 1,01 | 3,38 | 0,047 |
|  | 0.5 | 2,5 | 1,16 | 5,4 | 0,02 |
| ∆D1 _histBL | 0.1 | 1,17 | 0,78 | 1,74 | 0,45 |
|  | 0.2 | 1,14 | 0,76 | 1,71 | 0,53 |
|  | 0.3 | 1,2 | 0,79 | 1,81 | 0,39 |
|  | 0.4 | 1,28 | 0,84 | 1,95 | 0,24 |
|  | 0.5 | 1,25 | 0,81 | 1,93 | 0,31 |
| ∆D1 _estBL | 0.1 | 1,1 | 0,73 | 1,64 | 0,66 |
|  | 0.2 | 1,17 | 0,78 | 1,77 | 0,45 |
|  | 0.3 | 1,18 | 0,78 | 1,79 | 0,43 |
|  | 0.4 | 1,25 | 0,82 | 1,92 | 0,3 |
|  | 0.5 | 1,24 | 0,8 | 1,92 | 0,34 |

*∆H48h_histBL*= Serum creatinine increase based on the highest value during the first 48h after admission versus a historical baseline value, *∆H48h_ICUadm*=Serum creatinine increase based on the highest value during the first 48h after admission versus the ICU admission value, *∆H48h_estBL*=Serum creatinine increase based on the highest value during the first 48h after admission versus an estimated baseline value, *∆H24h_ICUadm*=Serum creatinine increase based on the highest value during the first 24h after admission versus the ICU admission value, *∆D1_histBL*=Serum creatinine increase based on the value on D1 (24h after admission) versus a historical baseline value, *∆D1_estBL*=Serum creatinine increase based on the value on D1 (24h after admission) versus an estimated baseline value.
